# The Emerging Role of Pattern Recognition Receptors in the Pathogenesis of Malaria

**DOI:** 10.3390/vaccines6010013

**Published:** 2018-02-28

**Authors:** Parisa Kalantari

**Affiliations:** Department of Immunology, Tufts University School of Medicine, Boston, MA 02111, USA; Parisa.Kalantari@tufts.edu; Tel.: +1-617-636-2979

**Keywords:** TLRs, malaria, innate immunity, Pattern recognition receptors, host-pathogen interaction, malaria immunity

## Abstract

Despite a global effort to develop an effective vaccine, malaria is still a significant health problem. Much of the pathology of malaria is immune mediated. This suggests that host immune responses have to be finely regulated. The innate immune system initiates and sets the threshold of the acquired immune response and determines the outcome of the disease. Yet, our knowledge of the regulation of innate immune responses during malaria is limited. Theoretically, inadequate activation of the innate immune system could result in unrestrained parasite growth. Conversely, hyperactivation of the innate immune system, is likely to cause excessive production of proinflammatory cytokines and severe pathology. Toll-like receptors (TLRs) have emerged as essential receptors which detect signature molecules and shape the complex host response during malaria infection. This review will highlight the mechanisms by which *Plasmodium* components are recognized by innate immune receptors with particular emphasis on TLRs. A thorough understanding of the complex roles of TLRs in malaria may allow the delineation of pathological versus protective host responses and enhance the efficacy of anti-malarial treatments and vaccines.

## 1. Introduction 

Malaria is one of the most devastating diseases of human history. In many developing countries, malaria remains to be a significant health burden with around one million deaths per year, mostly in children under the age of five [1,2]. *Plasmodium*, the causative agent of malaria, is an intracellular protozoan parasite, belonging to the genus *Plasmodium* and is transmitted by the bite of an infected female *Anophele* mosquito. Human malaria is caused by four species of *Plasmodium*: *P. falciparum, P. vivax, P. ovale, P. malariae*, with *P. falciparum* being the deadliest, causing cerebral malaria and anemia mostly in sub-Saharan Africa. A fifth species, *P. knowlesi*, which causes malaria in macaques, has also been reported to infect humans [3]. Although there has been a significant advance in the understanding of acquired immunity in malaria, the innate immune response to malaria has not been intensely scrutinized. An important aspect of the innate immune response is to trigger acquired immunity. Hence, understanding this area of malaria biology should prove critical in the search for an efficacious malaria vaccine.

Two major unresolved issues concerning the pathogenesis of malaria are: which components from the parasite activate the innate immune system to cause inflammation and, what are the host receptors that recognize these microbial products. Understanding the complexity of malaria pathogenesis is essential to developing strategies to control disease severity. In this review we focus on the interaction between host immune receptors (particularly TLRs) and malaria components, with the goal of understanding the host responses to *Plasmodium* and how the parasite modulates the immune system.

## 2. *Plasmodium* Life Cycle and Main Symptoms of the Infection

*Plasmodium* has a complex life cycle involving the human host and a mosquito vector. Infection is initiated by the bite of a female mosquito, Anopheles, which injects sporozoites into the skin. Sporozoites enter the bloodstream and invade hepatocytes in the liver (exoerythrocytic phase), where they undergo asexual replication and develop into thousands of liver stage merozoites, which are then released into the blood. This stage of infection is not associated with symptoms or tissue destruction. The released merozoites rapidly invade erythrocytes (erythrocytic phase) and multiply by asexual (mitotic) division. Inside the erythrocyte, merozoites develop into ring trophozoite and schizont stages. Mature schizonts rupture the host erythrocytes and release merozoites, which subsequently reinfect new erythrocytes. The release of merozoites coincides with the febrile paroxysm, which is highly characteristic of malaria. A small number of developing merozoites differentiate into male and female gametocytes. These gametocytes are ingested by the Anopheles mosquito during a blood meal. In the mosquito gut, the gametocytes differentiate into male and female gametes and fuse to make the zygote (ookinete), which forms an oocyst in the gut wall of Anopheles. Meiosis occurs in the oocyte, giving rise to sporozoites which travel to the salivary glands.

The main pathophysiological events during malaria infection are (a) erythrocyte destruction and ineffective erythropoiesis, (b) adhesion of *Plasmodium* infected red blood cells to capillary veins of vital host organs and (c) excessive production and the release of proinflammatory cytokines [4]. This cascade of events leads to fever, chills, rigors, vasodilatation, headache and excessive perspiration, i.e., the classical paroxysm [5].

## 3. Innate Immunity and Malaria

Innate immunity as the first line of defense has evolved multiple evolutionary conserved germline encoded pattern-recognition receptors (PRRs) to ensure detection and rapid response to a variety of pathogens. These receptors include Toll-like receptors (TLRs), retinoic acid inducible gene-I (RIG-I)- like receptors (RLRs) and nucleotide binding oligomerization domain (NOD)- like receptors (NLRs). These receptors activate distinct transcriptional programs and induce the synthesis of cytokines and chemokines not only to clear pathogens but also to induce the adaptive immune response. PRRs recognize conserved Pathogen-associated molecular patterns (PAMPs), which distinguish foreign organisms from self. PRRs reside in different cellular compartments. While TLRs detect PAMPs on the cell membrane of endosomes or the cell surface, RLRs and NLRs are cytosolic [6,7,8,9].

Innate immune responses begin in the early pre symptomatic stages of plasmodial infection, when the parasitemia is still very low. At this stage, innate immunity genes such as TLRs, PRRs and many proinflammatory cytokines are already upregulated [10]. Proinflammatory cytokines are upregulated in the serum of mice infected with various *Plasmodium* species at days 5–9 of infection [11]. Type I IFN is upregulated in the blood of malaria infected mice as early as 24–36 h postinfection [12]. Studies on human malaria revealed that the cytokine levels of TNFα, IFNγ and IL-12 in PBMCs infected with *Plasmodium falciparum* in vitro, remained elevated throughout the measurement period of 48 h after infection [13,14]. The profile of the proinflammatory cytokine responses appears to correlate with the pathophysiological and clinical symptoms of malaria [15]. The early proinflammatory response can lead to anemia, cerebral malaria and death [16,17]. Thus, the innate immune response appears to occur early and is apparently important in the ultimate outcome of the disease.

Immune cells such as macrophages and dendritic cells can discriminate between pathogens and self by their TLRs. A small number of TLRs are responsible for the recognition of a broad range of PAMPs. To date, 10 TLRs have been reported in humans and 13 in mice. Pathogen recognition by TLRs induces rapid activation of innate immunity by triggering the production of proinflammatory cytokines and the upregulation of co-stimulatory molecules [18]. Toll was originally discovered in *Drosophila melanogaster*, which has eight additional related receptors (Toll 2–9). TLRs have been reported to detect components derived from parasites such as *Plasmodium* [19,20,21,22,23]. Years of studies on malaria have shown a critical role for proinflammatory cytokines in pathogenesis of the disease [4,24] and high levels of proinflammatory cytokines such as TNFα, IL-6 and IFNγ have long been linked to acute phases of malaria, including cerebral malaria [4,24]. Mice deficient in adaptor molecule MyD88, demonstrate an impaired production of proinflammatory cytokines and a decrease in the intensity of symptoms, when infected with various *Plasmodium* strains [11,25,26]. Although the role of TLR2 and TLR4 in malaria remain unclear [25,27,28,29,30], mice deficient in TLR9 were reported to be resistant to PbA-induced experimental cerebral malaria (ECM), suggesting a pathological role of TLR9 rather than a protective role during cerebral malaria [11]. Therapy with E6446, a synthetic antagonist of nucleic-acid-sensing TLRs, was reported to diminish the activation of TLR9 and prevented the exacerbated cytokine response during cerebral malaria and the signs of ECM were prevented. This result supported the hypothesis that nucleic acid sensing TLRs are important in the development of ECM [31]. In addition to a potential direct role of TLR9 in the pathogenesis of cerebral malaria, a hyperresponsiveness of TLR9 in experimentally infected rodents was observed [32].

Major clinical symptoms in malaria patients are partly due to sequestration of infected red blood cells in the brain microvasculature. The sequestration is due to the binding of infected red blood cells to the host endothelial receptors such as intracellular adhesion molecule 1 (ICAM-1), vascular cellular adhesion molecule 1 (VCAM-1), endothelial/leukocyte adhesion molecule (ELAM-1) and CD36 [33]. The increase of proinflammatory cytokine release is responsible for the overexpression of adhesion molecules on brain endothelial cells causing the development of cerebral malaria [34]. Furthermore, cytokines such as TNFα and IFNγ which are released during malaria infection, suppress hematopoiesis and hence contribute to anemia, another main pathology of malaria [35,36]. Taken together, the excessive release of proinflammatory cytokines appears to be an important component of the pathogenesis of malaria. Although these types of observations might suggest that interference with TLR signaling might be beneficial in severely ill patients with malaria, the clinical experience with TLR inhibitors in other TLR mediated infectious diseases is not good. For example, a potent and non-toxic LPS inhibitor known as E5564 failed to reach the “primary endpoints” (improved morbidity and/or mortality) in human patients with gram-negative bacterial sepsis [37]. Yet, the role of LPS in gram-negative sepsis is well established. The most likely explanation for this failure is that anti-TLR drugs that are targeted towards acute infectious syndromes probably need to be administered to patients before they become ill.

## 4. Sensors of Plasmodial Components

Three components of the parasite have been studied in detail as potential immunomodulators during disease: (1) plasmodial glycosylphosphatidylinositol (GPI) anchors, (2) hemozoin and (3) plasmodial DNA. All of these molecules seem to be released during schizogeny and might contribute to the fever and chills that are the hallmark of malaria. In other words, each of these parasite components might be the “malaria toxin” that causes proinflammatory cytokine storm and potentially, death. We focus on recent studies that uncover the role of TLRs and other PRRs in the sensing of these molecules.

## 5. GPI Anchors

GPIs are heat stable and expressed on the cell surface of eukaryotes, where they anchor certain cell surface proteins to plasma membranes. GPIs are abundantly expressed in parasites, such as Trypanosoma, Leishmania, and *Plasmodium*. A relatively large pool of GPIs is shed, and can serve as a ligand for host innate immune receptors, inducing the production of proinflammatory cytokines [38]. Red blood cell lysis during the erythrocytic phase of *Plasmodium* life cycle results in the release of GPIs, which is recognized by TLR2-TLR6 or TLR1-TLR2 heterodimers and to a lesser extent by TLR4 homodimers [21,39,40]. GPI anchors induce TLR-mediated synthesis of proinflammatory cytokines such a TNFα and IL-1β [41], as well as nitric oxide [42] by host macrophages. GPI of malaria parasite is a candidate toxin which increases cell adhesion molecule expression via TLR activation resulting in a marked increase in parasite and leukocyte cytoadherence to endothelial cells [43]. Schofield et al. showed that anti-GPI vaccination prevents the development of pulmonary oedema, acidosis and cerebral malaria in *P. berghei* infection; although all immunized animals eventually succumbed to massive parasitemias, associated with a significant reduction in erythrocyte density [44]. Since immunized mice in this study were substantially protected against cerebral malaria and fatalities, anti-GPI vaccine seems promising, although the relevance of these observations in humans remains unclear.

## 6. Hemozoin

*Plasmodium* digests erythrocyte hemoglobin as a source of amino acids during the erythrocytic stage and generates high quantities of free toxic heme metabolites. Other organisms such as *Hemoproteus* parasites, *Schistosoma* and *Echinostoma* rediae *trivolis* and the insect *Rhodnius prolixus* also produce hemozoin [45,46,47]. Heme is capable of generating free radicals in the acidic digestive vacuole of the parasite. The parasite uses a heme detoxification mechanism that results in the formation of an insoluble, dark-brown crystal called hemozoin, formerly known colloquially as “malaria pigment”. Heme detoxification protein (HDP) has been identified to be secreted into red blood cell cytosol and transported with hemoglobin into the food vacuole of the parasite where hemozoin formation occurs. HDP binds to heme and converts it into hemozoin [48]. Malarial pigment has long fascinated researchers. In 1847, Johann Heinrich Meckel first observed hemozoin in the blood and spleen of a deceased patient who suffered from insanity. However, it was not until 1849 that the presence of the pigment was connected to infection with malaria [49]. As the parasite undergoes asexual replication in erythrocytes, schizont rupture results in the release and rapid phagocytosis of merozoites by immune cells such as macrophages and dendritic cells. With 1–10% parasitemia, 0.2–2 g hemozoin may be produced by *Plasmodium falciparum* during each cycle [50]. The release of free hemozoin, presumably from parasites that turn over during the replicative process, coincides with the induction of proinflammatory cytokines, including IL-1β and TNFα, and the periodic fevers characteristic of malaria [51,52]. Once released into the bloodstream, hemozoin becomes phagocytosed by immune cells in the spleen, liver and brain. The quantity of hemozoin released into the blood at schizogony has been suggested to reflect the parasite burden during malaria, and may be a measure of disease severity [53,54]; and, because the survival of the parasite depends on the formation of hemozoin, hemozoin is an appealing target for drug design. Many currently used anti-malarial drugs, such as chloroquine, are thought to prevent the polymerization of toxic heme released during proteolysis of hemoglobin by incorporating drug-heme complex into the growing polymer of hemozoin to end chain extension [55]. The prevention of heme detoxification and the subsequent accumulation of toxic heme in the food vacuole is deadly for the parasite.

Investigators use two distinct sources of hemozoin: that which they crystallize in the laboratory from hemin (“synthetic” hemozoin) and hemozoin extracted from parasite cultures (“natural” hemozoin). Natural hemozoin is bound by proteins, lipids and DNA [23,56,57]. The finding that attached molecules rather than the hemozoin crystal itself induce immunological responses, finds support in the strong adsorptive property of hemozoin [58]. Natural hemozoin contains abundant proteins noncovalently associated with the crystal [57,59,60,61,62] and the effects of these proteins remain to be fully defined. These proteins are from the host or the parasite and contribute to the hemozoin-mediated immune responses. Host fibrinogen is stably bound to hemozoin and strongly increases the capacity of natural hemozoin to activate monocyte inflammatory functions [63]. Protease treatment of natural hemozoin completely abolished its proinflammatory activity as reported previously [29,58,64,65]. Indeed, hemozoin, DNA and a host associated protein(s), including fibrinogen, could potentially form a complex and augment innate immune responses.

Hemozoin was initially reported to be the TLR9 ligand [22,66]. Coban et al., showed that natural and synthetic hemozoin bound to TLR9 directly in a manner that depends on particular motifs and amino acid sequences and induced conformational changes in the receptor; although the relevance of this interaction is not known [66]. However, it was subsequently found that the TLR9-stimulatory capacity of hemozoin is dependent on the presence of *Plasmodium* DNA, which is complexed to hemozoin and not to the hemozoin itself. Hemozoin acts as a carrier to transport plasmodial DNA into phagolysosomes where it can be sensed by TLR9 [23,67]. Hemozoin which is phagocytosed by macrophages is initially localized in the phagolysosomal compartment. At later time points, it is found in the cytosol. This movement of hemozoin from the phagolysosomal compartment to the cytosol highlights a mechanism by which hemozoin can facilitate the movement of plasmodial DNA into cytosol where it is recognized by nucleic acid sensors other than TLRs [67,68]. In fact, hemozoin and plasmodial DNA do colocalize in at least 40% of infected red blood cells [67]. One possibility is that during the process of parasite expansion, there is constant turnover of a subpopulation of parasites, and that during this process, hemozoin may be exposed to parasite DNA. Indeed, a recent paper by Love and colleagues [69,70] provides evidence that this occurs as the result of human platelet factor 4, which kills *Plasmodium* inside erythrocytes by selectively lysing the parasite digestive vacuole where hemozoin resides. This process was shown to liberate hemozoin from the food vacuole, ultimately killing the parasite. As the parasite nucleus undergoes karyolysis, it is clear that plasmodial DNA and hemozoin mix in the cytosol, where hemozoin has the opportunity to be bound by DNA.

The interaction of plasmodial DNA with TLR9 could lead to the development of new strategies for immunotherapy especially since TLR9 has also been known for its adjuvant potential [66]. TLR9 inhibitors have been developed for a variety of diseases such as systemic lupus erythematosus (SLE) [71], and block death in the mouse model of cerebral malaria [31]. Additionally, a role for hemozoin as a vaccine adjuvant has also been recently proposed. This adjuvant effect of synthetic hemozoin is TLR9 and NLRP3 independent and MyD88 dependent and its adjuvanticity depends on its size. Synthetic hemozoin with sizes between 50 and 200 nm had optimal adjuvant effects compared with larger molecules of synthetic hemozoin (2–20 μm). Natural hemozoin also has a potent adjuvant effect, independent of DNA but dependent on TLR9 [66]. 

The activation of inflammasome is broken down into two steps: a “priming” step, which is usually via the activation of TLRs and results in the production of pro-IL-1β and (b) a second step which results in the assembly of the inflammasome and generation of mature IL-1β [72]. It has been shown that the NLRP3 inflammasome senses crystalline materials, including hemozoin [73,74,75,76,77] but the mechanism by which hemozoin activates NLRP3 is unresolved. In all of the above studies, two experimental issues should be noted. First, as mouse macrophages do not express NLRP3 nor pro-IL-1β at rest, these studies relied upon pretreatment with LPS to “prime” inflammasome activity. Secondly, the source of the hemozoin crystals in all of these reports was “synthetic”. For both of these reasons, extrapolating from the in vitro data to the in vivo situation during malaria should be done with more than the usual amount of caution. We recently showed that natural hemozoin provides both signals for inflammasome activation. Natural hemozoin (by virtue of its CpG containing plasmodial DNA) induced TLR9 translocation, providing a priming signal for inflammasomes. After phagocytosis, hemozoin and DNA dissociate. Hemozoin subsequently induces phagolysosomal destabilization, allowing phagolysosomal contents access to the cytosol, where hemozoin activates NLRP3 inflammasome while plasmodial DNA activates AIM2 inflammasome, both providing the second signal for inflammasome activation. Therefore, as we learn more about inflammation in malaria, hemozoin has emerged as a key component of almost every aspect of innate immune activation. Based on these data, hemozoin does much more than help DNA engage TLR9, as it is essential for activation of NLRP3, AIM2 and even the type I IFN response that occurs in the cytosol of immune cells [67,68].

Nevertheless, it seems likely that hemozoin, even during disease, is an NLRP3 activator. Crystalline structures such as alum, silica, monosodium urate and cholesterol have been shown to destabilize phagolysosomes and release cathepsin B into cytosol [77,78]. Cathepsin activity appears to be involved in the activation of the NLRP3 inflammasome. Shio et al. and our group have found that hemozoin induced IL-1β production is dependent on cathepsin B [67,74], although using cathepsin B deficient cells, Dostert et al., failed to make a similar finding [75]. Griffith et al. have reported that hemozoin activates NLRP3 through the release of uric acid [73], yet Dostert et al. failed to reproduce this finding [75]. Similar to the results seen with other crystalline activators of NLRP3, intraperitoneal injection of hemozoin into mice has been reported to result in the recruitment of neutrophils to the peritoneal cavity, which is impaired in NLRP3, ASC, caspase-1 and IL-1R-deficient mice [73,74,75]. Shio et al. observed that Lyn and Syk are upstream of NLRP3 and required for its activation [74]. Intraperitoneal injection of hemozoin into mice has been reported to result in the recruitment of neutrophils to the peritoneal cavity, which is impaired in NLRP3, ASC, caspase-1 and IL-1R-deficient mice [67,72,73,74,75]. A possible explanation for all of these conflicting results might be different strains of *Plasmodium*, different routes of infection (for example by injection into peritoneal cavity of mice, the parasite bypasses the liver stage), various background of mice, various types and origins of phagocytic cells, various incubation times with hemozoin and various amounts and forms of hemozoin (synthetic and natural) prepared by different protocols.

The physiological significance of NLRs during malaria is controversial and a subject of debate. The major cause of this debate may be the inadequacy of the rodent models of the disease, which are especially troubling because neither the host nor the parasite are the same as that in any of the forms of malaria. Dorstet et al. clearly showed that the pathogenesis of cerebral disease is dampened in NLRP3 deficient mice infected with *Plasmodium berghei ANKA* sporozoites, while parasitemia remained unchanged [75]. This is in accordance with the data from Shio et al., showing that the absence of NLRP3 or IL-1β increased survival to *Plasmodium chabaudi adami DS* infection [74]. In contrast to these reports, a recent study suggests that components of inflammasome signaling are not involved in the pathogenesis of cerebral malaria and mice deficient in components of the inflammasome succumbed to infection with *Plasmodium berghei* ANKA with similar kinetics as wild type mice [76]. Similar results were reported by Kordes et al., ruling out a role for caspase-1 in experimental cerebral malaria (ECM) development [79]. In humans, many studies show a significant increase of proinflammatory cytokines such as IL-1β and IL-18 (e.g., [4,80,81]) and its association with fever episodes and severity of disease in symptomatic malaria patients [80,81]. NLRP3, AIM2 and NLRP12 inflammasomes appear to assemble in the PBMCs of patients with human malaria [82].

## 7. Plasmodial Nucleic Acids 

The significant role of nucleic acids as activators of innate immunity has been increasingly appreciated over the last decade. Many groups have reported that foreign DNA elicits an inflammatory response in cells. DNA can be detected in different cellular compartments and can induce a range of various cellular responses that are largely dependent on the subcellular compartment in which recognition takes place. TLR9 was discovered as the first receptor for hypomethylated CpG-rich DNA but it soon became clear that additional sensing mechanisms exist which led to the discovery of a diverse selection of intracellular DNA sensors. The list of DNA sensors includes DNA-dependent activator of interferon (IFN)-regulatory factors (DAI), absent in melanoma 2 (AIM2), RNA polymerase III (Pol III), leucine-rich repeat (in Flightless I) interacting protein-1 (Lrrfip1) DExD/H box helicases (DHX9 and DHX36) and the IFN-inducible protein IFI16 [reviewed in [83,84]].

CpG motifs in the plasmodial DNA has been shown to activate TLR9 in the endosomes and can stimulate AIM2 inflammasome activation when in cytosol [67]. Phagocytosis and digestion of *Plasmodium* infected red blood cells (RBCs) also release parasite DNA into phagolysosomes and subsequently into the cytosol, resulting in the activation of innate immune receptors. Additional reports suggest that plasmodial DNA is an important stimulatory constituent of the parasite that appears to be recognized by TLR9 [65,85]. Using extracts from cultured merozoites, Gowda et al. demonstrated that a protein-DNA complex was the main component from the parasite that activates dendritic cells through TLR9 [56]. A recent study shows that malaria patients have increased levels of circulating anti-DNA antibodies and circulating immunocomplexes (ICs) containing parasite DNA. Purified ICs trigger monocytes to produce high levels of proinflammatory cytokines [86]. It is possible that ICs bind to Fc receptors and get phagocytosed. When in the phagolysosomes, they release parasitic DNA which could be sensed by TLR9 and induce the production of proinflammatory cytokines. Additional evidence shows that *Plasmodium* infected red blood cells secrete extracellular vesicles containing plasmodial DNA and RNA. These vesicles will be internalized by monocytes, where plasmodial DNA is released in the cytosol and activates DNA sensing pathways [87].

The importance of DNA sensing and type I Interferon (IFN) release is now beginning to be appreciated in the context of various infectious diseases, beyond the traditional role of IFNα and IFNβ in antiviral immunity. Type I IFNs are typically generated in response to nucleic acids. A number of reports have shown that *Plasmodium* spp induce type I IFNs and present a type I gene signature [65,88,89,90,91]. In plasmocytoid dendritic cells, TLR9 is critical for the IFN response to schizonts [65]. Two recent publications showed that the production of type I IFN was dependent on TLR7 and not TLR9 in plasmocytoic DCs [12,91]. Daily intraperitoneal injections of a recombinant human IFN-α in mice infected with *P. berghei* ANKA (*PbA*) prevented death by cerebral malaria [89]. Polymorphisms in IFN-γ receptor are associated with malaria susceptibility [92,93] while polymorphisms in type I IFN receptor (*Ifnar1*) are associated with protection against cerebral malaria [88]. In addition to the role of plasmodial DNA to trigger TLR9, a novel TLR-independent DNA sensing pathway appears to be important in malaria recognition. This work is derived from the observation that *Plasmodium falciparum* DNA is extraordinarily AT-rich (AT-r), and contains a recurrent AT-r hairpin motif, ATTTTT(T)AC, over 6000 times. While the receptor for this DNA motif has yet to be identified, plasmodial AT-r motif is recognized by a cytosolic DNA sensing pathway involving STING, TBK1 and IRF3 leading to type I IFN production. Natural hemozoin that has been purified from *Plasmodium* cultures induced IFNβ and this effect was abolished after DNase treatment [68]. Mice deficient for the type I IFN receptor were almost completely protected from death, and mice deficient for IRF3 and IRF7 exhibited less severe symptoms than wild-type controls [12,68]. Based on these findings, AT-r motifs was proposed as a candidate PAMP. Recent work suggests that cGAS functions as a DNA sensor for detecting plasmodial DNA and triggering STING mediated type I IFN [12,91,94]. Yu et al. showed that STING diminishes TLR-dependent type I IFN responses via SOCS1 in pDCs during *P. yoelii* infection [91], while Gallego-Marin et al. showed that cGAS/STING pathway induces type I IFN in response to *P. falciparum* [94].

STING deficient mice were reported to be more resistant to malaria infection than the cGAS deficient mice, suggesting that additional DNA sensors might be involved in detecting plasmodial DNA [91]. Cytosolic RNA from *Plasmodium* has been reported to induce TLR7 [30,95] as well as melanoma differentiation-associated protein 5 (MDA5) and not RIG-I sensing pathway [91].

AIM2 is an inflammasome component that has been shown to directly recognize DNA and subsequently generate IL-1β. The AIM2 inflammasome is part of a larger family of inflammasomes, all of which are characterized by a Hind200 domain. AIM2 has clearly been demonstrated to be a sensor for DNA, and plays a role in the response to a number of microbes, including *Francisella tularensis*, vaccinia virus, mouse cytomegalovirus and *Listeria monocytogenes* [96,97,98,99]. Recent evidence suggest that AIM2 recognizes plasmodial DNA [67,86].

Nucleic acids also appear to be proinflammatory based on their degradation products. For example, *Plasmodium falciparum* infected erythrocytes have been found to accumulate uric acid, a major degradation product of purine metabolism. Both rodent and human data support the concept that urate, and possibly related degradation products, are proinflammatory in plasmodial infections [100,101,102].

## 8. TLR Polymorphism in Malaria

The evolution of the human genome has involved selective natural pressures imposed by various pathogens over the course of human history. Malaria has coexisted with humans for at least the last 60,000 years [103]. Considering its high infection rate, the disease has imposed strong evolutionary pressure on the genetic makeup of the innate immune system. Indeed, many gene mutations and polymorphisms that protect the host against severe malaria infection, such as the classic “balanced polymorphisms” associated with sickle cell trait [104], have been discovered in malaria. The parasite has also evolved over the many millennia of co habitation on earth and has found an ecological niche within the human erythrocyte that is nearly perfect for unhindered replication. This co adaptation of man with the parasite results in advantages for both host and parasite by reducing the probability of human death while simultaneously allowing for parasite spread.

Several examples of potentially important polymorphisms in innate immune genes that affect the outcome of malaria have been identified. These include, but are not limited to, CD40 ligand [105], nitric oxide syntase (NOS) [106,107] and TLR4 single nucleotide polymorphisms Asp299Gly and Thr399Ile [108,109]. These TLR4 single nucleotide polymorphisms (SNPs), as well as a Thr1486Cys polymorphism in TLR9, were associated with an increased risk of low birth weight and maternal anemia [110]. More recently, Chandy et al. [111] reported that two TLR9 SNPs are associated with higher levels of IFNγ in children with cerebral malaria, implying a causality to the association [111]. An additional fascinating observation was that of Khor and colleagues, who reported on a common SNP in the TLR adapter known as TIRAP (TIR domain containing adaptor protein) or Mal (MYD88 adaptor-like). This variant, MalSer180Leu, was associated with protection from death due to malaria in over 6000 patients from Gambia, Vietnam and Kenya [112], but only when present in hemizygosity. This SNP was also protective from several other pathogens including tuberculosis and *Streptococcus pneumoniae* [112]. The apparent protection of the MalSer180Leu SNP was thought to be due to an inability of the mutated protein to interact with the adapter MyD88, which is immediately downstream from Mal in the TLR2 and TLR4 signaling pathways. Hence, when present in hemizygosity, the authors believed the SNP had a partial anti-inflammatory effect [112]. Additional studies of this SNP have demonstrated that it is prevalent to widely varying degrees, depending on the population studied [113]. The Mal Ser180Leu SNP was also associated with mild disease in a GWAS study of malaria in Iranian patients [114]. Several studies have confirmed an apparent association with this variant gene in tuberculosis [115] and even Chagas disease [116].

## 9. TLRs as Adjuvants and Malaria Vaccine

The effort to develop an effective malaria vaccine has been ongoing for several decades now, and several large scale clinical studies have been performed. Indeed, part of the recent interest in the malaria field for innate immunity comes from the search for more potent and better tolerated adjuvants, which primarily work through the activation of innate immune responses. The oldest clinically approved adjuvant is alum, which is a general term to refer to any suspension of aluminum salts. Although alum activates the NLRP3 inflammasome, its adjuvant activity is not inflammasome dependent. Alternative theories about its mechanism of action abound (reviewed in [117]).

An emerging important new adjuvant is monophosphoryl lipid A (MPL), a TLR agonist with limited toxicity. MPL is thought to selectively activate the TRIF/TRAM pathway downstream from the TLR4/MD-2 receptor complex, and thus has reduced toxicity because it fails to fully activate an NF-κB pathway. MPL is approved as an adjuvant adsorbed onto aluminum hydroxide and is used in licensed vaccines against human papillomavirus and hepatitis B virus. Several large malaria vaccine studies have sought to exploit MPL as an adjuvant. The most recent study of the RTS,S/AS01 vaccine involved over 15,000 African children at risk for malaria. This vaccine study employed a recombinant chimeric protein consisting of the c-terminal portion of circumsporozoite antigen fused to hepatitis B surface antigen. “ASO1” refers to the adjuvant formulation consisting of a liposomal preparation containing MPL and QS21, a natural saponin triterpene glucoside that is purified from the bark of the soap bark tree, *Quillaja saponaria*. Despite the success of this vaccine trial, the developers of the vaccine hoped to see a far higher level of protection. TLR4-based adjuvants are likely to remain a top focus of these vaccine developers. Many other potential adjuvants are also under investigation including a variety of TLR-based immunomodulators and alternative activators of the innate immune system.

## 10. Conclusions

This review has attempted to update the immunology community on the most recent studies in the field of malaria innate immunity with special emphasis on the role of TLRs. Despite recent advances in control and elimination by the international community, malaria still remains a global burden with nearly half the planet’s population at risk for infection. Drug resistant strains of the parasite and insecticide-resistant mosquito vectors are a common problem, complicating public health measures. Understanding host sensing mechanisms, molecular pathways and cytokine responses is necessary for the development of an effective malaria vaccine.

## References

[1] Snow R.W., Guerra C.A., Noor A.M., Myint H.Y., Hay S.I. (2005). The global distribution of clinical episodes of *Plasmodium falciparum* malaria. Nature.

[2] Murray C.J., Rosenfeld L.C., Lim S.S., Andrews K.G., Foreman K.J., Haring D., Fullman N., Naghavi M., Lozano R., Lopez A.D. (2012). Global malaria mortality between 1980 and 2010: A systematic analysis. Lancet.

[3] Singh B., Kim Sung L., Matusop A., Radhakrishnan A., Shamsul S.S., Cox-Singh J., Thomas A., Conway D.J. (2004). A large focus of naturally acquired Plasmodium knowlesi infections in human beings. Lancet.

[4] Clark I.A., Budd A.C., Alleva L.M., Cowden W.B. (2006). Human malarial disease: A consequence of inflammatory cytokine release. Malar. J..

[5] Gazzinelli R.T., Kalantari P., Fitzgerald K.A., Golenbock D.T. (2014). Innate sensing of malaria parasites. Nat. Rev. Immunol..

[6] Dolasia K., Bisht M.K., Pradhan G., Udgata A., Mukhopadhyay S. (2018). TLRs/NLRs: Shaping the landscape of host immunity. Int. Rev. Immunol.

[7] Mogensen T.H. (2009). Pathogen recognition and inflammatory signaling in innate immune defenses. Clin. Microbiol. Rev..

[8] Brubaker S.W., Bonham K.S., Zanoni I., Kagan J.C. (2015). Innate immune pattern recognition: A cell biological perspective. Annu. Rev. Immunol..

[9] Takeuchi O., Akira S. (2010). Pattern recognition receptors and inflammation. Cell.

[10] Ockenhouse C.F., Hu W.C., Kester K.E., Cummings J.F., Stewart A., Heppner D.G., Jedlicka A.E., Scott A.L., Wolfe N.D., Vahey M. (2006). Common and divergent immune response signaling pathways discovered in peripheral blood mononuclear cell gene expression patterns in presymptomatic and clinically apparent malaria. Infect. Immun..

[11] Coban C., Ishii K.J., Uematsu S., Arisue N., Sato S., Yamamoto M., Kawai T., Takeuchi O., Hisaeda H., Horii T. (2007). Pathological role of Toll-like receptor signaling in cerebral malaria. Int. Immunol..

[12] Spaulding E., Fooksman D., Moore J.M., Saidi A., Feintuch C.M., Reizis B., Chorro L., Daily J., Lauvau G. (2016). STING-Licensed Macrophages Prime Type I IFN Production by Plasmacytoid Dendritic Cells in the Bone Marrow during Severe *Plasmodium yoelii* Malaria. PLoS Pathog..

[13] Scragg I.G., Hensmann M., Bate C.A., Kwiatkowski D. (1999). Early cytokine induction by *Plasmodium falciparum* is not a classical endotoxin-like process. Eur. J. Immunol..

[14] Artavanis-Tsakonas K., Riley E.M. (2002). Innate immune response to malaria: Rapid induction of IFN-gamma from human NK cells by live *Plasmodium falciparum*-infected erythrocytes. J. Immunol..

[15] Walther M., Woodruff J., Edele F., Jeffries D., Tongren J.E., King E., Andrews L., Bejon P., Gilbert S.C., De Souza J.B. (2006). Innate immune responses to human malaria: Heterogeneous cytokine responses to blood-stage *Plasmodium falciparum* correlate with parasitological and clinical outcomes. J. Immunol..

[16] Clark I.A., Rockett K.A. (1994). The cytokine theory of human cerebral malaria. Parasitol. Today.

[17] Haldar K., Mohandas N. (2009). Malaria, erythrocytic infection, and anemia. Hematology Am. Soc. Hematol. Educ. Program.

[18] Medzhitov R. (2001). Toll-like receptors and innate immunity. Nat. Rev. Immunol..

[19] Campos M.A., Almeida I.C., Takeuchi O., Akira S., Valente E.P., Procopio D.O., Travassos L.R., Smith J.A., Golenbock D.T., Gazzinelli R.T. (2001). Activation of Toll-like receptor-2 by glycosylphosphatidylinositol anchors from a protozoan parasite. J. Immunol..

[20] Gazzinelli R.T., Denkers E.Y. (2006). Protozoan encounters with Toll-like receptor signalling pathways: Implications for host parasitism. Nat. Rev. Immunol..

[21] Krishnegowda G., Hajjar A.M., Zhu J., Douglass E.J., Uematsu S., Akira S., Woods A.S., Gowda D.C. (2005). Induction of proinflammatory responses in macrophages by the glycosylphosphatidylinositols of *Plasmodium falciparum*: Cell signaling receptors, glycosylphosphatidylinositol (GPI) structural requirement, and regulation of GPI activity. J. Biol. Chem..

[22] Coban C., Ishii K.J., Kawai T., Hemmi H., Sato S., Uematsu S., Yamamoto M., Takeuchi O., Itagaki S., Kumar N. (2005). Toll-like receptor 9 mediates innate immune activation by the malaria pigment hemozoin. J. Exp. Med..

[23] Parroche P., Lauw F.N., Goutagny N., Latz E., Monks B.G., Visintin A., Halmen K.A., Lamphier M., Olivier M., Bartholomeu D.C. (2007). Malaria hemozoin is immunologically inert but radically enhances innate responses by presenting malaria DNA to Toll-like receptor 9. Proc. Natl. Acad. Sci. USA.

[24] Hunt N.H., Grau G.E. (2003). Cytokines: Accelerators and brakes in the pathogenesis of cerebral malaria. Trends Immunol..

[25] Franklin B.S., Rodrigues S.O., Antonelli L.R., Oliveira R.V., Goncalves A.M., Sales-Junior P.A., Valente E.P., Alvarez-Leite J.I., Ropert C., Golenbock D.T. (2007). MyD88-dependent activation of dendritic cells and CD4(+) T lymphocytes mediates symptoms, but is not required for the immunological control of parasites during rodent malaria. Microbes Infect..

[26] Adachi K., Tsutsui H., Kashiwamura S., Seki E., Nakano H., Takeuchi O., Takeda K., Okumura K., Van Kaer L., Okamura H. (2001). Plasmodium berghei infection in mice induces liver injury by an IL-12- and toll-like receptor/myeloid differentiation factor 88-dependent mechanism. J. Immunol..

[27] Gowda N.M., Wu X., Gowda D.C. (2012). TLR9 and MyD88 are crucial for the development of protective immunity to malaria. J. Immunol..

[28] Cramer J.P., Lepenies B., Kamena F., Holscher C., Freudenberg M.A., Burchard G.D., Wagner H., Kirschning C.J., Liu X., Seeberger P.H. (2008). MyD88/IL-18-dependent pathways rather than TLRs control early parasitaemia in non-lethal *Plasmodium yoelii* infection. Microbes Infect..

[29] Coban C., Ishii K.J., Horii T., Akira S. (2007). Manipulation of host innate immune responses by the malaria parasite. Trends Microbiol..

[30] Wu J., Tian L., Yu X., Pattaradilokrat S., Li J., Wang M., Yu W., Qi Y., Zeituni A.E., Nair S.C. (2014). Strain-specific innate immune signaling pathways determine malaria parasitemia dynamics and host mortality. Proc. Natl. Acad. Sci. USA.

[31] Franklin B.S., Ishizaka S.T., Lamphier M., Gusovsky F., Hansen H., Rose J., Zheng W., Ataide M.A., de Oliveira R.B., Golenbock D.T. (2011). Therapeutical targeting of nucleic acid-sensing Toll-like receptors prevents experimental cerebral malaria. Proc. Natl. Acad. Sci. USA.

[32] Franklin B.S., Parroche P., Ataide M.A., Lauw F., Ropert C., de Oliveira R.B., Pereira D., Tada M.S., Nogueira P., da Silva L.H. (2009). Malaria primes the innate immune response due to interferon-gamma induced enhancement of toll-like receptor expression and function. Proc. Natl. Acad. Sci. USA.

[33] Hisaeda H., Yasutomo K., Himeno K. (2005). Malaria: Immune evasion by parasites. Int. J. Biochem. Cell Biol..

[34] Gimenez F., Barraud de Lagerie S., Fernandez C., Pino P., Mazier D. (2003). Tumor necrosis factor alpha in the pathogenesis of cerebral malaria. Cell. Mol. Life Sci..

[35] Thawani N., Tam M., Stevenson M.M. (2009). STAT6-mediated suppression of erythropoiesis in an experimental model of malarial anemia. Haematologica.

[36] Lamikanra A.A., Theron M., Kooij T.W., Roberts D.J. (2009). Hemozoin (malarial pigment) directly promotes apoptosis of erythroid precursors. PLoS ONE.

[37] Opal S.M., Laterre P.F., Francois B., LaRosa S.P., Angus D.C., Mira J.P., Wittebole X., Dugernier T., Perrotin D., Tidswell M. (2013). Effect of eritoran, an antagonist of MD2-TLR4, on mortality in patients with severe sepsis: The ACCESS randomized trial. JAMA.

[38] Ropert C., Gazzinelli R.T. (2000). Signaling of immune system cells by glycosylphosphatidylinositol (GPI) anchor and related structures derived from parasitic protozoa. Curr. Opin. Microbiol..

[39] Nebl T., De Veer M.J., Schofield L. (2005). Stimulation of innate immune responses by malarial glycosylphosphatidylinositol via pattern recognition receptors. Parasitology.

[40] Durai P., Govindaraj R.G., Choi S. (2013). Structure and dynamic behavior of Toll-like receptor 2 subfamily triggered by malarial glycosylphosphatidylinositols of *Plasmodium falciparum*. FEBS J..

[41] Schofield L., Hackett F. (1993). Signal transduction in host cells by a glycosylphosphatidylinositol toxin of malaria parasites. J. Exp. Med..

[42] Zhu J., Krishnegowda G., Gowda D.C. (2005). Induction of proinflammatory responses in macrophages by the glycosylphosphatidylinositols of *Plasmodium falciparum*: The requirement of extracellular signal-regulated kinase, p38, c-Jun N-terminal kinase and NF-kappaB pathways for the expression of proinflammatory cytokines and nitric oxide. J. Biol. Chem..

[43] Schofield L., Novakovic S., Gerold P., Schwarz R.T., McConville M.J., Tachado S.D. (1996). Glycosylphosphatidylinositol toxin of Plasmodium up-regulates intercellular adhesion molecule-1, vascular cell adhesion molecule-1, and E-selectin expression in vascular endothelial cells and increases leukocyte and parasite cytoadherence via tyrosine kinase-dependent signal transduction. J. Immunol..

[44] Schofield L., Hewitt M.C., Evans K., Siomos M.A., Seeberger P.H. (2002). Synthetic GPI as a candidate anti-toxic vaccine in a model of malaria. Nature.

[45] Pisciotta J.M., Ponder E.L., Fried B., Sullivan D. (2005). Hemozoin formation in Echinostoma trivolvis rediae. Int. J. Parasitol..

[46] Biswas S., Karmarkar M.G., Sharma Y.D. (2001). Antibodies detected against *Plasmodium falciparum* haemozoin with inhibitory properties to cytokine production. FEMS Microbiol. Lett..

[47] Oliveira M.F., Silva J.R., Dansa-Petretski M., de Souza W., Lins U., Braga C.M., Masuda H., Oliveira P.L. (1999). Haem detoxification by an insect. Nature.

[48] Jani D., Nagarkatti R., Beatty W., Angel R., Slebodnick C., Andersen J., Kumar S., Rathore D. (2008). HDP-a novel heme detoxification protein from the malaria parasite. PLoS Pathog..

[49] Virchow R. (1849). Zur pathologischen des Bluts. Archiv fur Pathologische Anatomie und Physiologie und Klinische Medicin.

[50] Omodeo-Sale F., Motti A., Dondorp A., White N.J., Taramelli D. (2005). Destabilisation and subsequent lysis of human erythrocytes induced by *Plasmodium falciparum* haem products. Eur. J. Haematol..

[51] Sherry B.A., Alava G., Tracey K.J., Martiney J., Cerami A., Slater A.F. (1995). Malaria-specific metabolite hemozoin mediates the release of several potent endogenous pyrogens (TNF, MIP-1 alpha, and MIP-1 beta) in vitro, and altered thermoregulation in vivo. J. Inflamm..

[52] Kwiatkowski D., Nowak M. (1991). Periodic and chaotic host-parasite interactions in human malaria. Proc. Natl. Acad. Sci. USA.

[53] Nguyen P.H., Day N., Pram T.D., Ferguson D.J., White N.J. (1995). Intraleucocytic malaria pigment and prognosis in severe malaria. Trans. R. Soc. Trop. Med. Hyg..

[54] Amodu O.K., Adeyemo A.A., Olumese P.E., Gbadegesin R.A. (1998). Intraleucocytic malaria pigment and clinical severity of malaria in children. Trans. R. Soc. Trop. Med. Hyg..

[55] Sullivan D.J., Gluzman I.Y., Russell D.G., Goldberg D.E. (1996). On the molecular mechanism of chloroquine’s antimalarial action. Proc. Natl. Acad. Sci. USA.

[56] Wu X., Gowda N.M., Kumar S., Gowda D.C. (2010). Protein-DNA complex is the exclusive malaria parasite component that activates dendritic cells and triggers innate immune responses. J. Immunol..

[57] Goldie P., Roth E.F., Oppenheim J., Vanderberg J.P. (1990). Biochemical characterization of *Plasmodium falciparum* hemozoin. Am. J. Trop. Med. Hyg..

[58] Shio M.T., Kassa F.A., Bellemare M.J., Olivier M. (2010). Innate inflammatory response to the malarial pigment hemozoin. Microbes. Infect..

[59] Ashong J.O., Blench I.P., Warhurst D.C. (1989). The composition of haemozoin from *Plasmodium falciparum*. Trans. R. Soc. Trop. Med. Hyg..

[60] Kassa F.A., Shio M.T., Bellemare M.J., Faye B., Ndao M., Olivier M. (2011). New inflammation-related biomarkers during malaria infection. PLoS ONE.

[61] Jaramillo M., Bellemare M.J., Martel C., Shio M.T., Contreras A.P., Godbout M., Roger M., Gaudreault E., Gosselin J., Bohle D.S. (2009). Synthetic *Plasmodium*-like hemozoin activates the immune response: A morphology-function study. PLoS ONE.

[62] Goldberg D.E., Slater A.F. (1992). The pathway of hemoglobin degradation in malaria parasites. Parasitol. Today.

[63] Barrera V., Skorokhod O.A., Baci D., Gremo G., Arese P., Schwarzer E. (2011). Host fibrinogen stably bound to hemozoin rapidly activates monocytes via TLR-4 and CD11b/CD18-integrin: A new paradigm of hemozoin action. Blood.

[64] Coban C., Yagi M., Ohata K., Igari Y., Tsukui T., Horii T., Ishii K.J., Akira S. (2010). The malarial metabolite hemozoin and its potential use as a vaccine adjuvant. Allergol. Int..

[65] Pichyangkul S., Yongvanitchit K., Kum-arb U., Hemmi H., Akira S., Krieg A.M., Heppner D.G., Stewart V.A., Hasegawa H., Looareesuwan S. (2004). Malaria blood stage parasites activate human plasmacytoid dendritic cells and murine dendritic cells through a Toll-like receptor 9-dependent pathway. J. Immunol..

[66] Coban C., Igari Y., Yagi M., Reimer T., Koyama S., Aoshi T., Ohata K., Tsukui T., Takeshita F., Sakurai K. (2010). Immunogenicity of whole-parasite vaccines against *Plasmodium falciparum* involves malarial hemozoin and host TLR9. Cell Host Microbe..

[67] Kalantari P., DeOliveira R.B., Chan J., Corbett Y., Rathinam V., Stutz A., Latz E., Gazzinelli R.T., Golenbock D.T., Fitzgerald K.A. (2014). Dual engagement of the NLRP3 and AIM2 inflammasomes by plasmodium-derived hemozoin and DNA during malaria. Cell Rep..

[68] Sharma S., DeOliveira R.B., Kalantari P., Parroche P., Goutagny N., Jiang Z., Chan J., Bartholomeu D.C., Lauw F., Hall J.P. (2011). Innate immune recognition of an AT-rich stem-loop DNA motif in the *Plasmodium falciparum* genome. Immunity.

[69] Love M.S., Millholland M.G., Mishra S., Kulkarni S., Freeman K.B., Pan W., Kavash R.W., Costanzo M.J., Jo H., Daly T.M. (2012). Platelet factor 4 activity against P. falciparum and its translation to nonpeptidic mimics as antimalarials. Cell Host Microbe.

[70] McMorran B.J., Burgio G., Foote S.J. (2013). New insights into the protective power of platelets in malaria infection. Commun. Integr. Biol..

[71] Barrat F.J., Meeker T., Chan J.H., Guiducci C., Coffman R.L. (2007). Treatment of lupus-prone mice with a dual inhibitor of TLR7 and TLR9 leads to reduction of autoantibody production and amelioration of disease symptoms. Eur. J. Immunol..

[72] Stutz A., Golenbock D.T., Latz E. (2009). Inflammasomes: Too big to miss. J. Clin. Investig..

[73] Griffith J.W., Sun T., McIntosh M.T., Bucala R. (2009). Pure Hemozoin is inflammatory in vivo and activates the NALP3 inflammasome via release of uric acid. J. Immunol..

[74] Shio M.T., Eisenbarth S.C., Savaria M., Vinet A.F., Bellemare M.J., Harder K.W., Sutterwala F.S., Bohle D.S., Descoteaux A., Flavell R.A. (2009). Malarial hemozoin activates the NLRP3 inflammasome through Lyn and Syk kinases. PLoS Pathog..

[75] Dostert C., Guarda G., Romero J.F., Menu P., Gross O., Tardivel A., Suva M.L., Stehle J.C., Kopf M., Stamenkovic I. (2009). Malarial hemozoin is a Nalp3 inflammasome activating danger signal. PLoS ONE.

[76] Reimer T., Shaw M.H., Franchi L., Coban C., Ishii K.J., Akira S., Horii T., Rodriguez A., Nunez G. (2010). Experimental cerebral malaria progresses independently of the Nlrp3 inflammasome. Eur. J. Immunol..

[77] Hornung V., Bauernfeind F., Halle A., Samstad E.O., Kono H., Rock K.L., Fitzgerald K.A., Latz E. (2008). Silica crystals and aluminum salts activate the NALP3 inflammasome through phagosomal destabilization. Nat. Immunol..

[78] Duewell P., Kono H., Rayner K.J., Sirois C.M., Vladimer G., Bauernfeind F.G., Abela G.S., Franchi L., Nunez G., Schnurr M. (2010). NLRP3 inflammasomes are required for atherogenesis and activated by cholesterol crystals. Nature.

[79] Kordes M., Matuschewski K., Hafalla J.C. (2011). Caspase-1 activation of interleukin-1beta (IL-1beta) and IL-18 is dispensable for induction of experimental cerebral malaria. Infect. Immun..

[80] Gatton M.L., Cheng Q. (2002). Evaluation of the pyrogenic threshold for *Plasmodium falciparum* malaria in naive individuals. Am. J. Trop. Med. Hyg..

[81] Nagamine Y., Hayano M., Kashiwamura S., Okamura H., Nakanishi K., Krudsod S., Wilairatana P., Looareesuwan S., Kojima S. (2003). Involvement of interleukin-18 in severe *Plasmodium falciparum* malaria. Trans. R. Soc. Trop. Med. Hyg..

[82] Ataide M.A., Andrade W.A., Zamboni D.S., Wang D., Souza Mdo C., Franklin B.S., Elian S., Martins F.S., Pereira D., Reed G. (2014). Malaria-induced NLRP12/NLRP3-dependent caspase-1 activation mediates inflammation and hypersensitivity to bacterial superinfection. PLoS Pathog..

[83] Sharma S., Fitzgerald K.A. (2011). Innate immune sensing of DNA. PLoS Pathog..

[84] Hornung V., Latz E. (2010). Intracellular DNA recognition. Nat. Rev. Immunol..

[85] Gowda N.M., Wu X., Gowda D.C. (2011). The Nucleosome (Histone-DNA Complex) Is the TLR9-Specific Immunostimulatory Component of *Plasmodium falciparum* That Activates DCs. PLoS ONE.

[86] Hirako I.C., Gallego-Marin C., Ataide M.A., Andrade W.A., Gravina H., Rocha B.C., de Oliveira R.B., Pereira D.B., Vinetz J., Diamond B. (2015). DNA-Containing Immunocomplexes Promote Inflammasome Assembly and Release of Pyrogenic Cytokines by CD14+ CD16+ CD64high CD32low Inflammatory Monocytes from Malaria Patients. MBio.

[87] Sisquella X., Ofir-Birin Y., Pimentel M.A., Cheng L., Abou Karam P., Sampaio N.G., Penington J.S., Connolly D., Giladi T., Scicluna B.J. (2017). Malaria parasite DNA-harbouring vesicles activate cytosolic immune sensors. Nat. Commun..

[88] Aucan C., Walley A.J., Hennig B.J., Fitness J., Frodsham A., Zhang L., Kwiatkowski D., Hill A.V. (2003). Interferon-alpha receptor-1 (IFNAR1) variants are associated with protection against cerebral malaria in the Gambia. Genes Immun..

[89] Vigario A.M., Belnoue E., Gruner A.C., Mauduit M., Kayibanda M., Deschemin J.C., Marussig M., Snounou G., Mazier D., Gresser I. (2007). Recombinant human IFN-alpha inhibits cerebral malaria and reduces parasite burden in mice. J. Immunol..

[90] Voisine C., Mastelic B., Sponaas A.M., Langhorne J. (2010). Classical CD11c+ dendritic cells, not plasmacytoid dendritic cells, induce T cell responses to Plasmodium chabaudi malaria. Int. J. Parasitol..

[91] Yu X., Cai B., Wang M., Tan P., Ding X., Wu J., Li J., Li Q., Liu P., Xing C. (2016). Cross-Regulation of Two Type I Interferon Signaling Pathways in Plasmacytoid Dendritic Cells Controls Anti-malaria Immunity and Host Mortality. Immunity.

[92] Koch O., Awomoyi A., Usen S., Jallow M., Richardson A., Hull J., Pinder M., Newport M., Kwiatkowski D. (2002). IFNGR1 gene promoter polymorphisms and susceptibility to cerebral malaria. J. Infect. Dis..

[93] Naka I., Patarapotikul J., Hananantachai H., Tokunaga K., Tsuchiya N., Ohashi J. (2009). IFNGR1 polymorphisms in Thai malaria patients. Infect. Genet. Evol..

[94] Gallego-Marin C., Schrum J.E., Andrade W.A., Shaffer S.A., Giraldo L.F., Lasso A.M., Kurt-Jones E.A., Fitzgerald K.A., Golenbock D.T. (2018). Cyclic GMP-AMP Synthase Is the Cytosolic Sensor of *Plasmodium falciparum* Genomic DNA and Activates Type I IFN in Malaria. J. Immunol..

[95] Baccarella A., Fontana M.F., Chen E.C., Kim C.C. (2013). Toll-like receptor 7 mediates early innate immune responses to malaria. Infect. Immun..

[96] Rathinam V.A., Jiang Z., Waggoner S.N., Sharma S., Cole L.E., Waggoner L., Vanaja S.K., Monks B.G., Ganesan S., Latz E. (2010). The AIM2 inflammasome is essential for host defense against cytosolic bacteria and DNA viruses. Nat. Immunol..

[97] Fernandes-Alnemri T., Yu J.W., Juliana C., Solorzano L., Kang S., Wu J., Datta P., McCormick M., Huang L., McDermott E. (2010). The AIM2 inflammasome is critical for innate immunity to Francisella tularensis. Nat. Immunol..

[98] Warren S.E., Armstrong A., Hamilton M.K., Mao D.P., Leaf I.A., Miao E.A., Aderem A. (2010). Cutting edge: Cytosolic bacterial DNA activates the inflammasome via Aim2. J. Immunol..

[99] Kim S., Bauernfeind F., Ablasser A., Hartmann G., Fitzgerald K.A., Latz E., Hornung V. (2010). Listeria monocytogenes is sensed by the NLRP3 and AIM2 inflammasome. Eur. J. Immunol..

[100] Orengo J.M., Evans J.E., Bettiol E., Leliwa-Sytek A., Day K., Rodriguez A. (2008). Plasmodium-induced inflammation by uric acid. PLoS Pathog..

[101] Orengo J.M., Leliwa-Sytek A., Evans J.E., Evans B., van de Hoef D., Nyako M., Day K., Rodriguez A. (2009). Uric acid is a mediator of the *Plasmodium falciparum*-induced inflammatory response. PLoS ONE.

[102] Das B.S., Patnaik J.K., Mohanty S., Mishra S.K., Mohanty D., Satpathy S.K., Bose T.K. (1993). Plasma antioxidants and lipid peroxidation products in falciparum malaria. Am. J. Trop. Med. Hyg..

[103] Tanabe K., Mita T., Jombart T., Eriksson A., Horibe S., Palacpac N., Ranford-Cartwright L., Sawai H., Sakihama N., Ohmae H. (2010). *Plasmodium falciparum* accompanied the human expansion out of Africa. Curr. Biol..

[104] Ringelhann B., Hathorn M.K., Jilly P., Grant F., Parniczky G. (1976). A new look at the protection of hemoglobin AS and AC genotypes against plasmodium falciparum infection: A census tract approach. Am. J. Hum. Genet..

[105] Sabeti P., Usen S., Farhadian S., Jallow M., Doherty T., Newport M., Pinder M., Ward R., Kwiatkowski D. (2002). CD40L association with protection from severe malaria. Genes Immun..

[106] Burgner D., Usen S., Rockett K., Jallow M., Ackerman H., Cervino A., Pinder M., Kwiatkowski D.P. (2003). Nucleotide and haplotypic diversity of the NOS2A promoter region and its relationship to cerebral malaria. Hum. Genet..

[107] Burgner D., Xu W., Rockett K., Gravenor M., Charles I.G., Hill A.V., Kwiatkowski D. (1998). Inducible nitric oxide synthase polymorphism and fatal cerebral malaria. Lancet.

[108] Schroder N.W., Schumann R.R. (2005). Single nucleotide polymorphisms of Toll-like receptors and susceptibility to infectious disease. Lancet Infect. Dis..

[109] Mockenhaupt F.P., Cramer J.P., Hamann L., Stegemann M.S., Eckert J., Oh N.R., Otchwemah R.N., Dietz E., Ehrhardt S., Schroder N.W. (2006). Toll-like receptor (TLR) polymorphisms in African children: Common TLR-4 variants predispose to severe malaria. J. Commun. Dis..

[110] Mockenhaupt F.P., Hamann L., von Gaertner C., Bedu-Addo G., von Kleinsorgen C., Schumann R.R., Bienzle U. (2006). Common polymorphisms of toll-like receptors 4 and 9 are associated with the clinical manifestation of malaria during pregnancy. J. Infect. Dis..

[111] Sam-Agudu N.A., Greene J.A., Opoka R.O., Kazura J.W., Boivin M.J., Zimmerman P.A., Riedesel M.A., Bergemann T.L., Schimmenti L.A., John C.C. (2010). TLR9 polymorphisms are associated with altered IFN-gamma levels in children with cerebral malaria. Am. J. Trop. Med. Hyg..

[112] Khor C.C., Chapman S.J., Vannberg F.O., Dunne A., Murphy C., Ling E.Y., Frodsham A.J., Walley A.J., Kyrieleis O., Khan A. (2007). A Mal functional variant is associated with protection against invasive pneumococcal disease, bacteremia, malaria and tuberculosis. Nat. Genet..

[113] Hamann L., Kumpf O., Schuring R.P., Alpsoy E., Bedu-Addo G., Bienzle U., Oskam L., Mockenhaupt F.P., Schumann R.R. (2009). Low frequency of the TIRAP S180L polymorphism in Africa, and its potential role in malaria, sepsis, and leprosy. BMC Med. Genet..

[114] Zakeri S., Pirahmadi S., Mehrizi A.A., Djadid N.D. (2011). Genetic variation of TLR-4, TLR-9 and TIRAP genes in Iranian malaria patients. Malar. J..

[115] Castiblanco J., Varela D.C., Castano-Rodriguez N., Rojas-Villarraga A., Hincapie M.E., Anaya J.M. (2008). TIRAP (MAL) S180L polymorphism is a common protective factor against developing tuberculosis and systemic lupus erythematosus. Infect. Genet. Evol..

[116] Ramasawmy R., Cunha-Neto E., Fae K.C., Borba S.C., Teixeira P.C., Ferreira S.C., Goldberg A.C., Ianni B., Mady C., Kalil J. (2009). Heterozygosity for the S180L variant of MAL/TIRAP, a gene expressing an adaptor protein in the Toll-like receptor pathway, is associated with lower risk of developing chronic Chagas cardiomyopathy. J. Infect. Dis..

[117] Levitz S.M., Golenbock D.T. (2012). Beyond empiricism: Informing vaccine development through innate immunity research. Cell.

